# 

**Published:** 1846-03

**Authors:** 


### CONTENTS OF NO. III.

### ORIGINAL COMMUNICATIONS.

### ESSAYS AND CASES.

ART. I.—Letters from Europe. By JAMES C. CROSS, M.D., late Professor of the Institutes of Medicine in Transylvania University, 185
ART. II.—Cases treated in the Louisville Marine Hospital. Reported by *Mr. Chas. L. Lyle* and *Mr. W. A. Harris.* - - 196

### REVIEWS.

ART. III.—On Diseases of the Liver. By *George Budd, M.D.*, F.R.S., Professor of Medicine in King's College, London, and Fellow of Caius College, Cambridge. With colored Plates, and numerous Woodcuts. - - - - - - - - 201
ART. IV.—Phrenology Examined. By *P. Flourens*, Member of the French Academy, Perpetual Secretary of the Royal Academy of Sciences, (Institute of France), Member of the Royal Societies of London and Edinburg, of the Royal Academy of Sciences of Stockholm, of Munich, and of Turin, &c. &c. Professor of Comparative Physiology at the Natural History Museum at Paris. Translated from the second Edition of 1845, by *Charles De Lucena Meigs, M.D.*, Memb. Amer. Phil. Soc., &c. &c. Philadelphia: Hogan & Thompson. 1846. (16mo. pp. 144.) - - - - - - - - 225
ART. V.—Twenty-fifth Annual Report of the Bloomingdale Asylum for the Insane. For the year 1845. By *Pliny Earle, M.D.* Physician to the Asylum. Report of the Pennsylvania Hospital for the Insane. For the year 1845. By *Thomas S. Kirkbridge, M.D.*, Physician to the Institution. - - - - 232
ART. VI.—A treatise on Corns, Bunions, the Diseases of the Nails, and general management of the Feet. By *Lewis Durlacher*, Surgeon Chiropodist (by special appointment) to the Queen. Philadelphia: Lea & Blanchard. 1845. (12mo. pp. 134.) 237
ART. VII.—On the Theory and Practice of Midwifery. By *Fleetwood Churchill, M.D., M.R.I.A.*, Licentiate of the College of Physicians in Ireland; Physician to the Western Lying-in Hospital; Lecturer on Midwifery, &c., in the Richmond Hospital School of Medicine; author of "a Treatise on the Diseases of Females," &c. &c. With Notes and Additions, by *Robert M. Huston, M.D.*, Professor of Materia Medica and General Therapeutics, and formerly of Obstetrics and the Diseases of Women and Children, in the Jefferson Medical College of Philadelphia; President of the Philadelphia Medical Society, &c. &c. Second American edition. With one hundred and twenty-eight illustrations from drawings by Bagg and others. Engraved by Gilbert. Philadelphia: Lea & Blanchard. 1846. (8vo. pp. 525.) - - - - - - 240

Art. VIII.—An Elementary Treatise on Midwifery; or Principles of Tocology and Embryology. By *Alf. A. S. M. Velpeau, M.D.*, &c &c. Translated from the French, by *Charles D. Meigs, M.D.*, Member of the American Philosophical Society, &c. &c, Third American edition. With Notes and Additions. By *William Harris, M.D.*, Lecturer on Midwifery and the Diseases of Women and Children, &c. &c. Philadelphia: Lindsay & Blackiston. 1846. (8vo. pp. 800.) - - 242

Art. IX.—A Manual of Chemistry. By *Richard D. Hoblyn A.M.* Oxon. Author of a Dictionary of Terms used in Medicine and the Collateral Sciences. New York: Samuel S. & William Wood. 1846. (18mo. pp. 335.) - - - - - 242

Art. X.—Elements of Surgery. By *Robert Liston*, Surgeon to the North London Hospital; &c. &c. Fourth American from the last London edition; with copious Notes and additions: By *Samuel D. Gross, M.D.*, Professor of Surgery in the Medical Institute of Louisville, &c. &c. Illustrated with numerous engravings. Philadelphia: Ed. Barrington & Geo. D. Haswell; Louisville, Ky.: James Maxwell. 1846. (8vo. pp. 664.) 243

Art. XI.—Lectures on the Operations of Surgery, and on Diseases and Accidents requiring operations. By *Robert Liston, Esq.*, F.R.S., Senior Surgeon to the University College Hospital, &c. With numerous Additions. By *Thomas D. Mütter, M.D.*, Professor of Surgery in Jefferson Medical College, Philadelphia, &c. &c. &c. Philadelphia: Lea & Blanchard. 1846. (8vo. pp. 565.) - - - - - - - 244

SELECTIONS FROM AMERICAN AND FOREIGN JOURNALS.

Contagiousness of Puerperal Fever, - - - - - - 245
Sub-carbonate of Iron in the treatment of Uterine Hemorrhage, 246
Phosphate of Ammonia, in Gout and Rheumatism, - - - 252
Lithotripsy, - - - - - - - - - - 253
On the effects of Food on the Blood, - - - - - 255
Longings of Pregnant Women, - - - - - - 256
Headache, - - - - - - - - - - 256
Observations on Acid Rain, - - - - - - - 257
Gonorrhœa, - - - - - - - - - - 257
The Brocchieri Styptic, - - - - - - - 258
Honor to Bichat, - - - - - - - - - 258
Death of medical men in Boston, - - - - - - 259
On the treatment of Bilious Colic by copious enemata, - - 259
Gunshot wound of the Brain, - - - - - - - 261
Expulsion and extraction of the placenta before the child, - 264
Infantile diseases depending upon Inflammation, - - - 267
Statistics of suicide in France, - - - - - - 268

ORIGINAL INTELLIGENCE.

Kentucky Lunatic Asylum, - - - - - - - - 269
Latin Prescriptions,—The Brocchieri Styptic, - - - 271
Memphis medical school,—Transylvania University, - - 273
Facts from Correspondents, - - - - - - - - 274
Meteorological Table. - - - - - - - - - 276

